# Breeding Habitat Preferences of Major *Culicoides* Species (Diptera: Ceratopogonidae) in Germany

**DOI:** 10.3390/ijerph17145000

**Published:** 2020-07-11

**Authors:** Doreen Werner, Sarah Groschupp, Christian Bauer, Helge Kampen

**Affiliations:** 1 Research Area 2 “Land Use and Governance”, Leibniz Centre for Agricultural Landscape Research (ZALF), 15374 Müncheberg, Germany; sarah.groschupp@zalf.de; 2 Working Group “Epidemiology”, Institute of Parasitology, Justus Liebig University, 35392 Giessen, Germany; christian.bauer@vetmed.uni-giessen.de; 3 Institute of Infectology, Friedrich-Loeffler-Institut, Federal Research Institute for Animal Health, 17493 Greifswald, Germany; helge.kampen@fli.de

**Keywords:** bluetongue, breeding sites, *Culicoides*, biting midge, dung, Germany, habitat, Obsoletus Complex, Schmallenberg disease, vector management

## Abstract

Biting midges of the genus *Culicoides* (Diptera, Ceratopognidae) are demonstrably or putatively involved in the transmission of both bluetongue (BTV) and Schmallenberg viruses (SBV) in Central Europe. Although these insects are ubiquitous in Europe, relatively little is known about their requirements in terms of breeding habitats and substrates. *Culicoides* species composition and relative abundance in potential breeding habitats were therefore studied at various locations in Northeastern Germany and one location in Western Germany by emergence trap collections. Forty-three potential breeding sites were analyzed in ten landscape structures, with 28,091 adult biting midges emerging from them. Among these, 2116 specimens belonged to the genus *Culicoides*. Species of the culicoid subgenus *Avaritia* were most abundant (70.6% of all specimens) and widespread (91.6% of all sites), while the subgenus *Culicoides* accounted for 15.6% of the specimens registered but emerged from 70.8% of all sites sampled. *Culicoides* species of other subgenera were collected in 75.0% of all studied sites, with a relative abundance of 8.7%. The results indicate that various types of dung, but probably also some landscape habitats, offer suitable substrates for the development of potential *Culicoides* vector species. Adaptations in dung management on farms and landscape design and use might therefore be appropriate approaches to reduce the risk of BTV or SBV transmission.

## 1. Introduction

Due to their painful bites and often high abundances, female biting midges of numerous species of the genus *Culicoides* are well-known pests affecting humans and livestock [1]. In addition, some species are vectors of disease agents [2]. In Central and Northern Europe, the transmission of pathogens by biting midges was regarded as exotic until the advent of bluetongue disease (BT) in 2006 [3]. Globalization and modern transportation technologies involving long-distance animal movement facilitate the travel of both infected vertebrates and vectors to reach almost any place in the world within a short time [4]. Thus, when animals infected with a vector-borne disease agent are imported, indigenous competent vectors, which may ingest and replicate the pathogen, pose a significant risk of local transmission. In the case of small insects like biting midges, passive drift by wind over hundreds of kilometres is discussed, too, allowing for biting midge-borne pathogens to be introduced via their vectors [5].

Soon after the onset of the BT epidemic in Central Europe, it became clear that the Old World’s major vector of BT virus (BTV), *Culicoides imicola* Kieffer, 1913 had not spread from its Mediterranean distribution areas to the affected regions. Instead, European *Culicoides* species belonging to the Obsoletus Complex (subgenus *Avaritia*) and the Pulicaris Complex (subgenus *Culicoides*) were strongly suspected as candidates for virus transmission and have been incriminated as potential BTV vectors in Europe [3,6,7]. BTV has now repeatedly been demonstrated in field collections of these species [8], but evidence from experimental transmission is still lacking, not the least because these species cannot be reared in the laboratory. The large geographic distribution over much of Europe of most of the species, which tested virus-positive, could explain the rapid spread of BT between 2006 and 2009 as well as that of the subsequent Schmallenberg disease from 2011 to 2013 [9], and it may support future outbreaks of culicoid-borne diseases in this region [10].

In contrast to the adults of many *Culicoides* species, the females of which are traditionally collected by UV-light traps, scarce knowledge exists on the preimaginal developmental stages. Little is known, for example, about the breeding habitat selection and adaptation of the various species, and, in many cases, their preimaginal stages and their breeding sites are completely unknown [11].

Generally, culicoid larvae and pupae are supposed to develop in wet and humid habitats of all types, where they can be found in the top layer of the ground [12,13]. These habitats range from bogs over wetlands to saltwater marshes [14]. Additionally, numerous species have been demonstrated in aquatic zones of flowing and stagnant waters [15,16,17]. Some species, however, seem to avoid flooded areas [18] and prefer dung habitats [19,20,21,22], while still others use compost for immature development [23,24]. The further characterization of these habitats may provide important clues about the species composition of the respective biotopes.

Adults are usually found close to the larval habitats. In contrast to many other nematocerans, biting midges are considered poor flyers and have limited activity ranges. Actively, they rarely cover distances of more than 3 km [25].

All stages of biting midges are sensitive to environmental changes. Thus, they are strongly temperature-sensitive, and, depending on the ambient temperature, the completion of their development takes between a few days and several weeks or even months in winter [26,27].

The BT epidemic in 2006–2009 caused by BTV serotype 8 (BTV-8) was finally controlled by vaccination [28], whereas the Schmallenberg disease outbreak in 2011–2012 probably subsided due to herd immunity [29]. While Schmallenberg virus (SBV) never completely disappeared from Central Europe [30,31], BTV-8 re-emerged in France in 2015 and has since kept circulating [32], with new cases in Germany in 2018 [33].

Since diseases, including vector-borne ones, may nowadays emerge and resurge at any time and in any place in the world thanks to globalization, targeted disease control, e.g., by vaccination, can only be accomplished with delay. Vector control, however, is independent of the specific disease as long as the vector species are known. Therefore, vector populations should be managed where, when and if possible, be it proactively to prevent outbreaks or reactively during an outbreak, as a supplementary measure. This strategy is generally pursued with mosquitoes and mosquito-borne diseases, where mosquito larval stages and habitats are the first targets of intervention [34,35]. A prerequisite for this approach, however, is a fundamental knowledge of the biology and breeding characteristics of the vector species, which is scarce regarding *Culicoides* biting midges.

To contribute to unveiling the breeding habitat and substrate specialization of culicoids, data from various emergence trap collection studies targeting biting midges were analyzed. The results were expected to provide a better understanding of the ecological requirements of the immature developmental stages of potential *Culicoides* vectors and, consequently, of distribution areas and barriers which are caused by the presence or absence of breeding habitats. Such information could help predict the occurrence of biting midges in selected biotopes and improve risk assessments of culicoid-borne diseases.

## 2. Materials and Methods

### 2.1. Study Sites and Trapping Procedure

**Figure 1:**
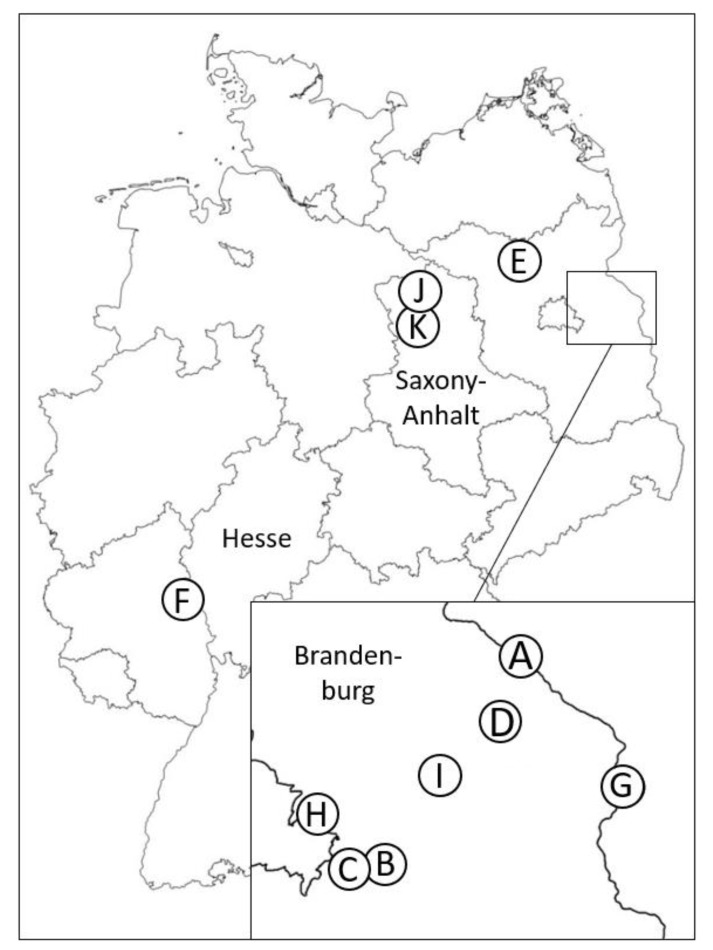
Geographical allocation of study regions sampled for emerging adult biting midges. A: Two locations with seven study sites; B: Two locations with three study sites; C: Two locations with three study sites; D: Four locations with six study sites; E: Two locations with four study sites; F: One location with two study sites; G. One location with two study sites; H: One location with six study sites; I: Five locations with seven study sites; J: One location with two study sites; K: One location with one study site.

Freshly emerged adult biting midges were collected in Western and Northeastern Germany by emergence traps, from May to December 1994, 2005 and 2007–2012 (Table S1). The traps were located in ten wet or humid landscape structures, such as river banks and floodplains, along the rivers Oder, Spree and Rhin, in the reed belts of lakes, wetlands and grasslands (both with and without bushes and trees), swampy forests and renaturation areas. The latter were only represented by floodplains along small streams. These areas had once been converted to forests for wood production and to greenlands for silage production and have only recently been restored to their natural status as floodplains. Further traps were positioned in specific “habitats”: on liquid manure, compost and different types of dung (two to three days old), on animal holdings and on meadows and pastures with animal husbandry in Oderbruch floodplain areas in the German federal states of Brandenburg and Saxony-Anhalt (Figure 1). All collection sites had a damp underground.

In addition to “landscape structure” and “habitat”, the terms “(study) region”, “location” and “(study) site” are used in this contribution. The “study region” refers to the greater geographical region where the study was performed, as depicted in Figure 1. A “study region” can include several “locations”, which are smaller administrative units specified by the local name. Finally, a “study site” defines the exact place where an emergence trap was operated.

A detailed compilation of the geographic regions, locations, study sites, numbers of used traps and landscape structures, as based on the CORINE (Coordination of Information on the Environment) land cover index but modified for small-scale assessments, is provided in Table S1. CORINE land cover indices were used to relate land cover and use to *Culicoides* occurrence.

Traps of 1 × 1 m^2^ ground area were mostly used, except for some dung and liquid manure substrates, where traps of 0.5 × 0.5 m^2^ were used, based on the habitat dimensions. All data collected were converted to the 0.5 × 0.5 m^2^ scale to facilitate comparison between collection sites.

Insects emerging from the soil covered by the traps were collected in plastic containers mounted on top of the traps and filled with 70% ethanol to kill and preserve the material. All traps were run once per month for 48 h.

At site 29 (River Oder, Güstebieser Loose), in the floodplains of the river Oder, site 31 (River Spree, Mönchwinkel), in the floodplains of the river Spree, and site 34 (Backwater River Oder, Platkow), on the banks of a backwater of the river Oder, emergence traps were set up on meadows where cattle, sheep or domestic ducks were maintained. For comparison, emergence traps were placed at three sites (sites 1, 2, 3) in the same region on floodplains without livestock and contamination by excreta. Like other floodplains of lowland rivers sampled in the study (sites 5, 7), these regions are characterized by open grasslands and dominated by abandoned agricultural meadows.

In addition to these field areas, eleven farms in the same region were included, according to the keeping of livestock producing various types of dung (Table S1). For setting the traps, both homogeneous and mixed types of dung were selected. As for the latter, mixed sheep/horse and goat/horse dung could be studied in Kiehnwerder (site 32) and mixed cattle/horse dung in Rosenthal (site 33).

Soil pH values were measured under each emergence trap using a Multi 3410 measuring device (WTW, Germany), and they were analyzed using SPSS 24.0 statistical software (IBM, Amonk, NY, USA).

### 2.2. Biting Midge Identification

*Culicoides* biting midges were sorted from the catches and morphologically identified mostly to species level or to a lower taxonomic resolution when this was not possible, using various Diptera-specific identification keys [36,37,38,39,40].

Within the subgenus *Avaritia*, the females of the Obsoletus Complex (*C. obsoletus* (Meigen, 1818)*, C. scoticus* (Downes and Kettle, 1952)) cannot be morphologically distinguished. Additionally, the identification of *C. chiopterus* (Meigen, 1830) and *C. dewulfi* (Goetghebuer, 1935) is challenging, particularly so when caught by emergence traps. Firstly, specimens often become conserved in the fixative before hardening of the chitinous parts and acquiring their final coloration. Secondly, samples exposed to the sunlight, as was the case in the present study due to the transparent construction material of the collection boxes of the emergence traps used, tend to bleach. Therefore, only a fraction of the *Avaritia* females could be identified to species level. Thus, the focus was on males, as these can be reliably identified to species by their genitalia. If males were not available, randomly selected females were processed by species-specific diagnostic PCR assays [41,42]. Some culicoids were subjected to COI barcoding using primers LCO1490 and HCO2198 (Eurofins Genomics, Ebersberg, Germany) [43] or primers PanCuli-COX1-211F and PanCuli-COX1-727R (Eurofins) [42] for reliable species identification.

### 2.3. Data Analysis 

Data were analyzed in R version 3.5.2 (R Foundation, Vienna, Austria) [44] using the vegan package [45]. Non-metric multidimensional scaling (NMDS) was used to plot the relative habitat preferences of the subgenera *Avaritia* and *Culicoides* and of the group of species belonging to other subgenera, as well as of species of subgenera other than *Avaritia*. The subgenus *Avaritia* was not considered in the second analysis, since only a fraction of the females was identified to species level and unidentified females could have a major impact on the data analysis should they not display the same species ratio as that of the identified specimens. To harmonize the collection data, the analysis was based on the average number of emergent culicoid specimens per trap and year. The suitability of the data was demonstrated by a stress test before applying the NMDS, using the Bray–Curtis similarity index with 100 iterations for reaching a stable solution.

## 3. Results

In 2160 48-h collections, more than one million insects were caught by emergence trap sampling, the majority (79.4%) of which belonged to dipteran species. In addition to Sphaeroceridae (43%), Psychodidae (14%), Sciaridae (7.0%), Sepsidae (4.3%), Chironomidae (3.1), Stratiomyiidae (1.8%) and Ceratopogonidae (0.5%), other families such as Drosophilidae, Muscidae, Cecidomyiidae and Calliphoridae were registered with low specimen numbers. Family and species diversity varied depending on landscape and breeding habitat, although 91.8% of all insects were collected from dung.

In single 48-h catches, the percentage of Ceratopogonidae varied from 0.1% to 32%. Many ceratopogonid specimens belonged to the genus *Forcipomyia* and to further genera other than *Culicoides* (Table 1 and Table 2). Culicoids were found in 817 collections (37.8%).

Considering individual landscapes and habitats, the total number of emerged culicoids varied between very few or even single specimens per 48 h in swampy forest areas (sites 16, 17) or liquid manure habitats (sites 42, 43) to more than 50 in renaturation areas (site 6) and dung sites on farms (sites 32, 33).

Twenty-one species of the genus *Culicoides* were identified. In total, the proportion of biting midges of this genus accounted for 0.2% (2116 specimens) of the total insect catches. These included all four species of the subgenus *Avaritia* occurring in Germany (*C. obsoletus*, *C. scoticus, C. chiopterus*, *C. dewulfi*). Furthermore, four members of the subgenus *Culicoides* were found: *C. delta* (Edwards, 1939), *C. pulicaris* (Linnaeus, 1758), *C. punctatus* (Meigen, 1804) and *C. newsteadi* (Austen, 1921), with the latter three grouped into the Pulicaris Complex. The remaining *Culicoides* species belonged to subgenera other than *Avaritia* or *Culicoides* (Table 1).

Based on the male *Avaritia* and *Culicoides* specimens (female specimens were not included due to the *Avaritia* females not being reliably identified to species level), 92.6% of *Culicoides* specimens belonged to the *Avaritia* subgenus and 6.7% to the *Culicoides* subgenus. *Culicoides obsoletus* and *C. scoticus* as members of the Obsoletus Complex were particularly abundant (83.3% and 10.4% of all *Avaritia* males, respectively), while *C. chiopterus* (5.2%) and *C. dewulfi* (1.1%) males were found in low numbers. In the subgenus *Culicoides*, *C. delta* accounted for most of the collected specimens (37.1% including females).

Subgenera were differently represented with respect to landscapes and habitats (Table 1). Species of the subgenus *Avaritia* were predominant in most landscape structures, such as floodplains (67.3% of all culicoids), meadows (80.0%), reed belts (65.7%) and boglands (78.2%). In “compost” and “dung” habitats, they reached a particularly high share of 96.3% and 88.7%, respectively, while no *Avaritia* specimens were found in renaturation areas or liquid manure. Species of the subgenus *Culicoides* were common in the “renaturation area/floodplain” landscape (93.9% of all culicoids) but emerged in small numbers only from almost all other studied types of landscapes and habitats, i.e., floodplains (22.7%), meadows (19.4%), grassland/wetlands (32.4%), banks of rivers and lakes (10.5%), swampy forests (1.4%), reed belts (32.5%), boglands (13.9%), compost (3.6%) and dung (2.5%) (Table 1).

In “mixed grassland/wetland with bushes and trees”, subgenus *Culicoides* specimens accounted for 33.4%, while the species of the subgenus *Avaritia*, which was represented by the Obsoletus Complex only, accounted for 41.9%, and species of other subgenera (*C. achrayi, C. fagineus, C. minutissimus, C. nubeculosus*) for 24.5% of the culicoid biting midges. Banks of rivers and lakes seemed to be attractive for members of the subgenus *Avaritia* (*C. obsoletus, C. scoticus, C. chiopterus*), which made up 47.6%, while individuals of other subgenera (*C. achrayi, C. circumscriptus, C. fagineus, C. kibunensis*) reached 41.8% and those of the subgenus *Culicoides* (*C. pulicaris, C. punctatus*) reached 10.4% of all culicoid biting midges collected. Single individuals of three species of other subgenera, *C. nubeculosus, C. riethi* and *C. festivipennis*, were found in liquid manure.

**Figure 2:**
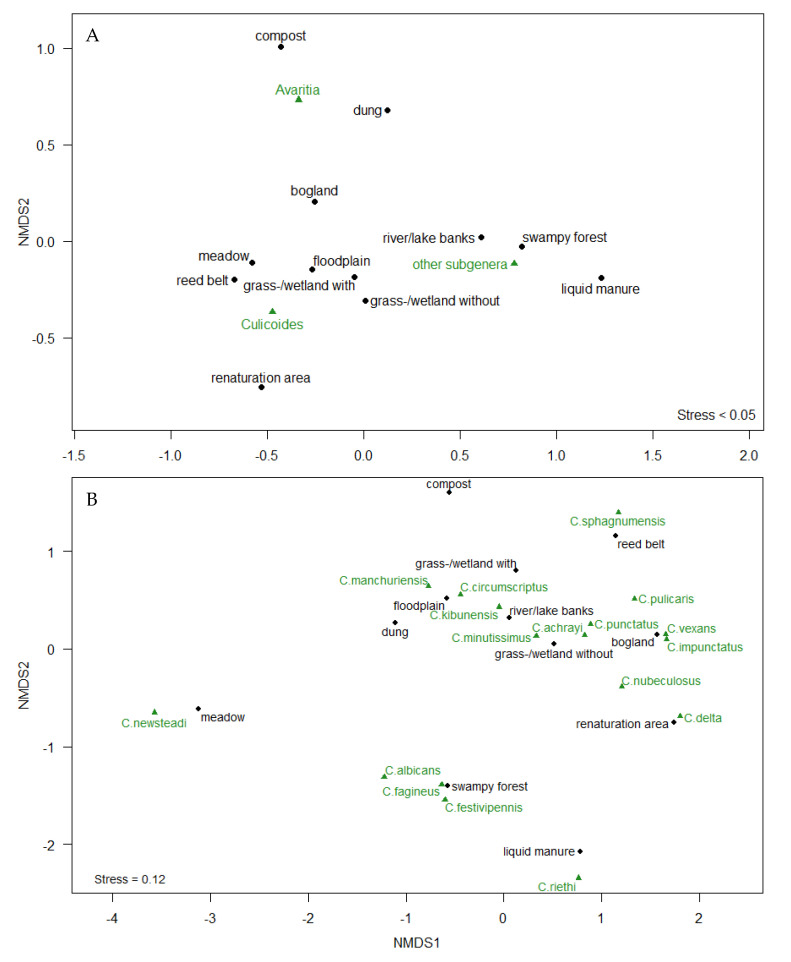
Two-dimensional NMDS ordination plot of habitat preference by culicoid subgenus/group (**A**) and species (**B**—subgenus *Avaritia* excluded for species). Distance of habitats (black points) to biting midge subgenus and species (green triangles) are a measure of habitat preference. Abbreviations of landscape/habitat types: grass-/wetland without—mixed grassland/wetland without bushes and trees; grass-/wetland with—mixed grassland/wetland with bushes and trees.

According to the NMDS analysis, which calculates a relative habitat preference (i.e., the result for one subgenus/species depends on the consideration of all other subgenera/species), the subgenus *Avaritia* was strongly associated with compost and dung habitats and the subgenus *Culicoides* with wetland, grassland, floodplain, meadows, mixed grassland/wetland with and without bushes and trees and reed belt landscapes, and the group of species belonging to other subgenera was associated with swampy forests and the banks of rivers and lakes (Figure 2A). In this analysis, it should be taken into account that the grouping eliminates ecological differences between species, which is particularly relevant with regard to the species belonging to the subgenera *Avaritia* and *Culicoides* as these are systematically more mixed and might be only distantly related.

When individual sites are considered, the highest numbers of *Culicoides* species and specimens emerged from dung sites (sites 24, 29, 33, 36) and a renaturation area/floodplain (site 6) and the lowest number from meadows (site 5). In the subgenus *Avaritia*, high numbers of specimens were obtained from dung habitats (sites 32, 33, 36), floodplains (site 1) and compost (sites 24, 26), and low numbers were obtained from swampy forests (sites 16, 17) and boglands (site 23). The highest numbers of subgenus *Culicoides* specimens were collected in renaturation areas (site 6), mixed grassland/wetland with bushes and trees (sites 9, 11) and reed belts (site 21), and low numbers were collected from swampy forests (site 16) and meadows (site 4).

The highest number of a species of the subgenus *Avaritia*, *C. obsoletus*, was collected from cowpats and llama/alpaca dung (sites 29, 36), while in the subgenus *Culicoides*, *C. delta* reached the highest collection numbers in a renaturation area (site 6). Among the species belonging to other subgenera, *C. achrayi* was the most abundant, namely in mixed grassland/wetland with and without bushes and trees (sites 12, 14, 15) and on the banks of rivers (site 21).

The NMDS analysis for landscape/habitat preferences of species (except for *Avaritia* species) shows *C. sphagnumensis* to be most closely associated with reed belts; *C. manchuriensis* and *C. circumscriptus* with floodplains; *C. kibunensis* with the banks of rivers and lakes; *C. minutissimus* with mixed grasslands/wetlands without bushes and trees and the banks of rivers and lakes; *C. achrayi* with mixed grassland/wetland without bushes and trees; *C. punctatus* with mixed grassland/wetland without bushes and trees and boglands; *C. vexans* and *C. impunctatus* with boglands; *C. pulicaris* with boglands and reed belts; *C. nubeculosus* with mixed grassland/wetland without bushes and trees and renaturation areas; *C. delta* with renaturation areas; *C. riethi* with liquid manure; *C. albicans, C. fagineus and C. festivipennis* with swampy forests and *C. newsteadi* with meadows (Figure 2B).

Special attention was given to the study of dung as a potential breeding substrate, as species of the subgenus *Avaritia* are supposed to preferentially develop in organic-rich material [46]. Accordingly, pure dung substrates, where *Avaritia* species in fact predominated, produced twice as many specimens as the studied landscape habitats, with a particularly clear preference of both species of the Obsoletus Complex, *C. obsoletus* and *C. scoticus*, as well as *C. chiopterus* for dung habitats (Table 1). Depending on the origin of the dung, differences in the abundance and composition of the various emerging species became apparent (Table 2).

*Culicoides obsoletus* was mainly found to emerge from decaying cowpats, the stacked dung of cattle, llamas and alpacas and all types of mixed dung. The latter also served as substrates for *C. dewulfi* and *C. scoticus.* In addition, *C. scoticus* was collected from cowpats and the dung of sheep and horses, whereas *C. chiopterus* and *C. dewulfi* were found in rotten cattle dung as well as in dung of mixed composition. *Culicoides chiopterus* developed quite frequently in cowpats and stacked cattle dung, horse dung and, to a lesser extent, in mixed sheep/horse dung, but also in duck feces. In summary, *C. scoticus* seems to be the *Avaritia* species that is least adapted to a specific kind of dung. *Culicoides dewulfi* was found at two of the studied dung sites only. It was the only species developing in chicken dung. Lower numbers of species, but higher numbers of specimens, of the subgenus *Avaritia* were recovered from llama and alpaca dung as compared to cattle or mixed dung.

For dung substrates, numbers of emerged specimens of the subgenus *Culicoides* were also much lower than those of the subgenus *Avaritia*, and no individuals of the subgenus *Culicoides* at all emerged from llama, alpaca, chicken and pig dung. Of *C. pulicaris* and *C. punctatus*, only single individuals could be found in rabbit, deer, sheep and duck dung and mixed goat/horse dung, and only a few more were found in mixed sheep/horse dung.

An NMDS test according to dung type was not possible due to insufficient data (stress value too small).

**Figure 3:**
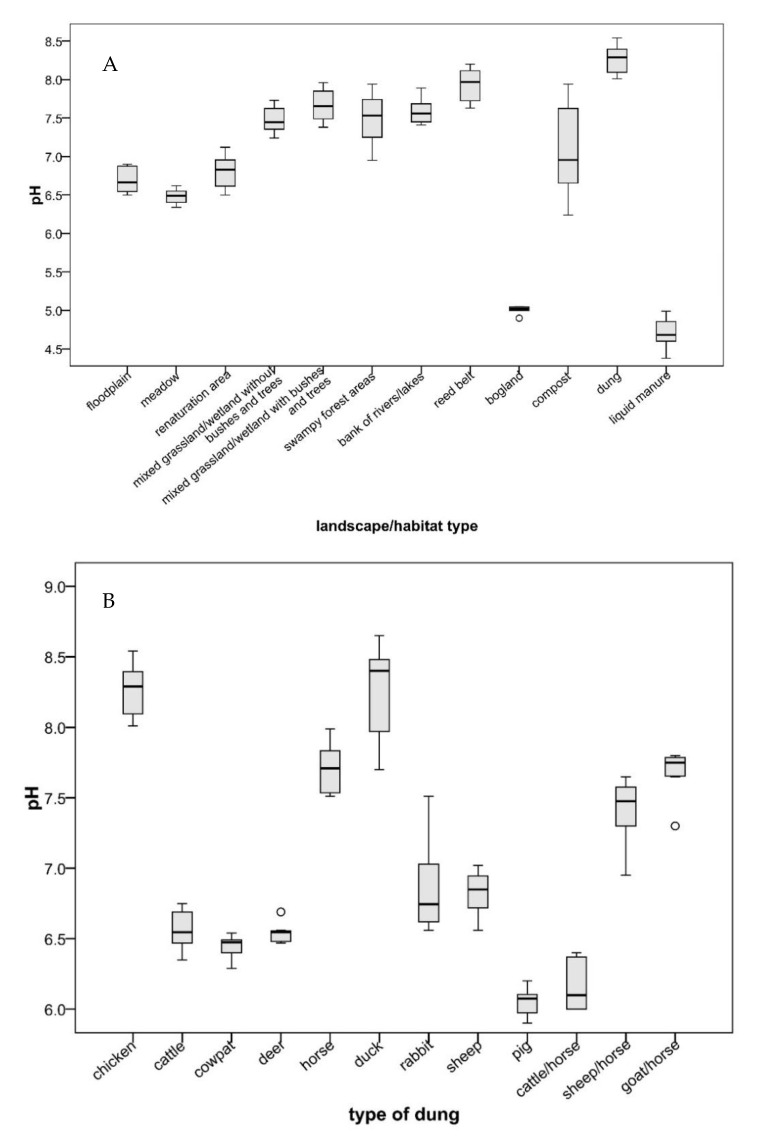
Box plots of pH values of the studied landscape/habitat types (**A**) and the types of dung (**B**), showing median, 25th and 75th percentiles and minimum and maximum values. Circles indicate outliers.

Muddy substrates used in renaturation areas, swampy forest areas, floodplains or meadows along streams were found to have a neutral pH value (Figure 3A). By contrast, substrates from grassland/wetland and compost habitats (rotten vegetables) were slightly alkaline, while samples from dung were strongly alkaline. Overall, the higher occurrence probability of *Avaritia* and *Culicoides* species was associated with neutral or low pH values (Figure 3A). The unexpected collection of culicoids from chicken, duck and pig dung indicates their potential to adapt to extreme breeding habitats with higher pH values (Figure 3B).

A total of 27.5% of the *Culicoides* samples collected by the emergence traps were males, 36.0% within the subgenus *Avaritia*, 16.6% within the subgenus *Culicoides* and 1.0% within other subgenera of the genus *Culicoides*. The highest percentage of *C. obsoletus* males was caught in September 2008 from a cattle dung heap, the highest percentage of *C. chiopterus* males in May 2008 from cowpats, the highest percentage of *C. scoticus* males in June and August 2009 from mixed cattle/horse dung and the highest percentage of *C. dewulfi* in May and September 2009 from cattle dung. In the Pulicaris Complex, the highest numbers of *C. pulicaris* and *C. punctatus* males were caught in floodplain areas and mixed grassland/wetland covered with bushes and trees in May and June 2008 and 2009, respectively.

## 4. Discussion

Despite recent extensive research on biting midge vectors, data on habitat binding and substrate preference by the various *Culicoides* species are still widely missing. One reason may be that data are almost impossible to generate by standardized methods and on a quantitative basis, since most biting midge species cannot be reared in the laboratory. Field data on breeding site productivity can rarely be quantitative and compared between locations and regions, even if the breeding sites are thoroughly characterized. Most likely, several additional unknown factors that determine productivity, not least the general composition of an area’s biting midge fauna, are not taken into account when data are analyzed. Even with UV-light traps, commonly used for collecting adult biting midges, it is not possible to assess an area’s general species composition satisfactorily, since not all species are attracted to these traps [47].

Moreover, UV-light traps commonly used for collecting adult culicoids [48,49,50,51,52] provide no information on *Culicoides* breeding sites or substrates. The above-mentioned limitations were partially overcome in the present study with the use of emergence traps, enabling the detection of *Culicoides* species freshly emerging from their developmental substrates. The advantages over manually collecting larvae from their substrates are manifold. Firstly, emerged adults can be morphologically identified much more easily than larvae or pupae, which are mostly difficult to identify or even unidentifiable at species level. Secondly, the emergence method is surface-related and can at least be standardized in this regard as the caught insects emerge from a well-defined area of the ground surface. Thirdly, it is not truly known how deep larvae wander into the ground in different habitats (e.g., under cowpats). Collecting potential breeding substrates from the ground surface might therefore miss developmental stages from below. Finally, the emergence trap technique is not invasive, so the soil biocenosis is not disturbed.

The study also showed that the CORINE land cover system, which is based on squares of 25 ha, is not appropriate for assigning potential breeding sites to landscape structures. Areas that are suitable or unsuitable as *Culicoides* breeding habitats differ on a much smaller scale. For the purpose of data analysis, the landscape structures in which traps were placed were therefore manually adjusted to real land use.

So far, 333 species of Ceratopogonidae have been described for Germany, with 57 of them belonging to the genus *Culicoides* [53,54]. In the present investigation, 21 *Culicoides* species were found in different German federal states by emergence traps, but the species diversity between sampling habitats and sites differed strongly. Some of the collected species seem to be ubiquitous, others to have a very limited and local distribution or to occur sympatrically with other species.

Species of the Obsoletus and Pulicaris Complexes occur almost worldwide [36] and are found all over Europe, whereby the taxonomic assignment of species to complexes (or “groups”) is unclear and inconsistently practiced, resulting in tremendous difficulties in biological analyses [55,56]. *Culicoides dewulfi*, for example, is traditionally considered a member of the Obsoletus Complex due to its morphological similarity to *C. obsoletus* and *C. scoticus*, but its taxonomic status is still controversially discussed [57,58].

In the subgenus *Avaritia*, most of the female specimens collected in the present study were not identified to species level. The correlation between species and substrate was done on the morphological identification of males and the genetic identification of selected females. According to this, *C. obsoletus*, followed by *C. chiopterus* and *C. scoticus*, seems to be the species of the subgenera *Avaritia*, which accepts the most diverse breeding substrates. In the present investigation, these three species were caught at almost all sampling sites, especially often in floodplains, wetlands and the silting zones of lakes, and they were generally extremely abundant in all types of dung accumulations. González et al. [18] found species of the Obsoletus Complex in different types of manure and rotten organic material, while both Ninio et al. [20] and Thompson et al. [59] collected Obsoletus Complex specimens predominantly from cattle dung. Although Zimmer et al. [60] also detected *C. obsoletus* and *C. scoticus* in silage, these findings suggest that the dung of vertebrates is an important breeding habitat for the species of the subgenus *Avaritia*.

Regarding the variety of breeding habitats used, a similar situation is true for the subgenus *Culicoides*, which seems to occur ubiquitously in Germany [61,62,63]. Although the number of collected specimens of the subgenus *Culicoides* (mainly *C. pulicaris* and *C. punctatus*) was much lower than the number of specimens of the subgenus *Avaritia* in the present study, these species were found in almost all studied habitats, e.g., floodplains, grassland/wetland, banks of lakes, boglands and dung substrates (except manure). Thompson et al. [60] also noted species of the Pulicaris Complex in various types of dung, while González et al. [18] found *C. punctatus* to commonly develop in the mud of pond margins. In the present study, *C. deltus* developed in the same habitats as *C. pulicaris* and *C. punctatus* but, as opposed to those, had extremely high densities in renaturation areas. *Culicoides newsteadi* was found in meadows only.

Apart from the subgenus *Avaritia*, the most abundant and widespread group in the present study, all *Culicoides* species, including those of the Pulicaris Complex, were collected in small numbers. This may suggest that *Culicoides* species not belonging to the subgenus *Avaritia* are much more specialized and stenoecious regarding eco-climatic conditions. Although the data do not allow a founded phenological analysis, the collection data seem to suggest that the general emergence of culicoid species seems to peak twice annually, in May–June and in August–September. Differences in the abundance of the species of the subgenus *Avaritia* and of other subgenera occurred throughout the season, which is in agreement with the findings of Saerle et al. [64,65]. The species composition varied considerably between the sampled sites, with a clear tendency of numbers of species and specimens increasing with humidity and portion of decaying organic matter. It remains unclear, however, whether the low emergence rate in some habitats was a result of a low oviposition rate and, as a consequence, a low larval occurrence or of a combination of other biotic and/or abiotic factors.

The species *C. achrayi*, *C. circumscriptus*, *C. festivipennis* and *C. nubeculosus* are known to breed in organic-rich matter [66]. Hence, it is not surprising that these species were demonstrated in or alongside floodplains, river banks, reed belts, boglands and swampy areas. A similar investigation from Turkey [67] showed *C. circumscriptus* and *C. festivipennis* to be common in comparable habitats, while Uslu and Dik [68] found *C. festivipennis*, *C. punctatus* and *C. brunnicans* particularly often in mud at pond margins.

Observations that neutral or low pH values of substrates were associated with a higher occurrence probability of species of the subgenera *Avaritia* and *Culicoides* in contrast to alkaline substrates were already made by Steinke et al. [21] for *C. obsoletus*, *C. chiopterus* and *C. dewulfi* reared from cowpats. In this study, high pH values were also registered for the breeding substrates of *C. nubeculosus* and *C. festivipennis*. Both species had previously been found in slushy dung next to natural water reservoirs characterized by alkaline pH values [68,69]. Blackwell et al. [70] did not find any relationship between larval density and soil pH value for *C. impunctatus* in woodland and coastal habitats.

The present study tried to correlate landscape structures/habitats and breeding sites to subgenus and species, although the latter was not possible within the subgenus *Avaritia* due to the uncertain species assignment of the majority of females collected. The analysis shows a wide variability of breeding options accepted by many species, with a certain species-specific tendency of preferences. However, the data are still preliminary and should be supplemented by further studies.

## 5. Conclusions

Numerous studies have demonstrated that it is extremely difficult to define the requirements of biting midge species by their developmental substrates since their breeding habitats are widely unknown. Thus, even the identification of single emerging adults can be helpful to specify the adaptation of species.

Breeding habitats of putative *Culicoides* vector species, in which specimens develop in significant numbers, were identified for the subgenera *Avaritia*, mainly the Obsoletus Complex, in the form of dung substrates. Preferences of species in dung selection could be detected depending on the origin and the quality of the dung. Thus, in order to control the main vectors of BTV and SBV in Northern Europe which are considered to belong to the Obsoletus Complex [71,72], it may be an important step to properly manage dung deposition, storage and processing on farms. The breeding habitat preferences of other species including secondary vectors might affect landscape design and use.

Although this study has expanded the knowledge of habitats and dung substrates as *Culicoides* breeding sites, the selection and ecology of these are far from being defined. Further studies providing more detailed knowledge about the breeding sites and microhabitats of *Culicoides* biting midges in different landscape structures are urgently needed for the risk assessment of culicoid-borne diseases and targeted vector control.

## Figures and Tables

**Table 1:** Emergence of biting midge subgenera and species according to landscape/habitat type (adjusted to a covered ground area of 0.5 x 0.5 m^2^), based on both qualitative and quantitative data from the years 1994, 2005 and 2007–2012 (females/males; +—present, −—no record; ^1^—on lakes; ^2^—on rivers; ^3^—on liquid cattle manure; ^4^—on liquid sheep manure; numbers in parentheses represent the number of genetically identified females).

Species	Landscape/Habitat Type											
	Floodplain	Meadow	Renaturation Area/Floodplain	Mixed Grassland/Wetland without Bushes and Trees	Mixed Grassland/Wetland with Bushes and Trees	Swampy Forest Areas	Banks of Rivers and Lakes	Reed Belt	Bogland	Compost	Dung	Liquid Manure
Genus *Culicoides* Subgenus *Avaritia* (Fox, 1955)												
*C. chiopterus* (Meigen, 1830)	+(3)/5	+(2)/2	–	+/2	–	+(4)/1	+/2	+/3 ^1^	–	+/2	+(3)/11	–
*C. obsoletus* (Meigen, 1818)	+(1)/20	+/9	–	+(1)/9	+(2)/4	–	+(2)/10	+(2)/8 ^1^	+(1)/8	+(4)/32	+(5)/354	–
*C. scoticus* (Downes and Kettle, 1952)	+(2)/10	–	–	+(5)/8	+/4	–	+/5	+(4)/0 ^1^	–	–	+(1)/23	–
*C. dewulfi* (Goetghebuer, 1935)	+(1)/2	–	–	–	–	–	–	–	+/1	–	+/3	–
Total number of females/males belonging to subgenus *Avaritia*	31/37	17/11	0/0	22/19	81/8	23/1	24/17	64/11	81/9	46/34	567/391	0/0
Genus *Culicoides* Subgenus *Culicoides* (Latreille, 1809)												
*C. delta* (Edwards, 1939)	1/1	–	116/8	–	1/0	–	–	–	2/0	–	5(1)/1	–
*C. pulicaris* (Linnaeus, 1758)	2/1	–	–	18(3)/0	24(3)/2	1/0	2/0	32/5 ^1,2^	5(1)/6	3/0	2(8)/0	–
*C. punctatus*(Meigen, 1804)	14(2)/2	–	–	1/2	35(2)/4	–	4/3	–	1/1	–	8(1)/0	–
*C. newsteadi* (Austen, 1921)	–	(4)/3	–	–	–	–	–	–	–	–	(1)/0	–
Total number of females/males belonging to subgenus *Culicoides*	19/4	4/3	116/8	22/2	65/6	1/0	6/3	32/5	9/7	3/0	26/1	0/0
Genus *Culicoides* Species of other subgenera												
*C. achrayi* (Kettle and Lawson, 1955)	–	–	3/0	15/1	31(2)/0	–	27/0	1/0 ^1^	1/0	–	74/0	–
*C. albicans* (Winnertz, 1852)	–	(1)/0	–	–	–	1/0	–	–	–	–	16/0	–
*C. circumscriptus* (Kieffer, 1918)	4(2)/0	–	–	–	–	–	2(1)/0	–	–	–	–	–
*C. fagineus* (Edwards, 1939)	–	–	–	–	11/0	13/1	4/0	–	–	–	1/0	–
*C. festivipennis* (Kieffer, 1914)	–	–	1/0	–	–	31/2	–	–	–	–	–	(1)/0 ^4^
*C. impunctatus* (Goetghebuer, 1920)	–	–	(2)/0	–	–	–	–	–	2/0	–	–	–
*C. kibunensis* (Tokunaga, 1937)	–	–	–	–	–	–	(1)/0	–	–	–	–	–
*C. manchuriensis* (Tokunaga, 1941)	(2)/0	–	–	–	–	–	–	–	–	–	(1)/0	–
*C. minutissimus*(Zetterstedt, 1855)	–	–	–	–	(1)/0	–	–	–	–	–	(2)/0	–
*C. nubeculosus*(Meigen, 1930)	2/0	–	–	–	7/0	–	–	–	1/0	–	1/0	2/0 ^3,4^
*C. riethi* (Kieffer, 1914)	–	–	–	–	–	–	–	–	–	–	–	(1)/0 ^4^
*C. sphagnumensis* (Williams, 1955)	–	–	–	–	–	–	–	1/0 ^2^	–	–	–	–
*C. vexans* (Staeger, 1839)	–	–	2/0	–	–	–	–	–	5/0	–	–	–
Total number of females/males of other subgenera	10/0	1/0	8/0	15/1	52/0	45/3	35/0	2/0	9/0	0/0	95/0	4/0
*Atrichopogon* spec.	–	–	–	–	+	–	–	+ ^1,2^	–	+	–	–
*Forcipomyia* spec.	+	–	–	+	+	+	+	+ ^1,2^	+	+	+	+
*Dasyhelea* spec.	–	–	–	–	–	–	–	–	–	–	+	–
*Palomyia* spec.	–	–	–	–	–	–	–	+ ^1^	+	–	–	–
*Serromyia* spec.	–	–	–	–	–	–	–	–	+	–	–	–

**Table 2:** Emergence of biting midge subgenera and species from various types of dung (adjusted to a covered ground area of 0.5 x 0.5. m^2^), based on both qualitative and quantitative data from the years 1994, 1995 and 2007–2012 (females/males; +—present, −—no record; numbers in parentheses represent the number of genetically identified females).

Species	Dung type												
	Llama/Alpaca	Chicken	Cattle	Cowpat	Deer	Horse	Duck	Rabbit	Sheep	Pig	Mixed Cattle/Horse	Mixed Sheep/Horse	Mixed Goat/Horse
Genus *Culicoides* Subgenus *Avaritia* (Fox, 1955)													
*C. chiopterus* (Meigen, 1830)	–	–	+(1)/43	+(2)/21	+/2	+(2)/19	+/4	–	–	–	–	+(2)/27	–
*C. obsoletus* (Meigen, 1818)	(89)/9	–	+(4)/50	+/10	–	+(1)/8	–	+/5	+(1)/13	+(3)/0	+(1)/24	+/8	+(2)/24
*C. scoticus* (Downes and Kettle, 1952)	(1)/6	–	–	+/2	+(2)/5	+/0	–	–	+(1)/9	–	+(2)/7	+/16	+(3)/0
*C. dewulfi* (Goetghebuer, 1935)	–	+/4	+/18	+/28	–	–	–	+/4	+/4	–	+(1)/0	+/3	+/18
Total number of females/males belonging to subgenus *Avaritia*	90/15	8/4	56/111	34/61	12/7	65/27	13/4	21/9	21/26	3/0	57/31	58/54	129/42
Genus *Culicoides* Subgenus *Culicoides* (Latreille, 1809)													
*C. delta* (Edwards, 1939)	–	–	–	–	–	–	–	–	(1)/0	–	–	–	–
*C. pulicaris* (Linnaeus, 1758)	–	–	−	−	(2)/0	–	–	1/0	3/0	–	–	(1)/1	1/0
*C. punctatus*(Meigen, 1804)	–	–	–	–	–	–	5/0	-	–	–	−	6/0	–
*C. newsteadi* (Austen, 1921)	–	–	−	–	–	(6)/0	–	–	–	–	–	–	–
Total number of females/males belonging to subgenus *Culicoides*	0/0	0/0	0/0	0/0	2/0	6/0	5/0	1/0	4/0	0/0	0/0	7/1	1/0
Genus *Culicoides* Species of other subgenera													
*C. achrayi* (Kettle and Lawson, 1955)	50(1)/0	–	–	–	–	–	–	–	8/0	–	–	–	–
*C. albicans*(Winnertz, 1852)	(16)/0	–	–	–	–	–	–	–	–	–	–	–	–
*C. fagineus* (Edwards, 1939)	–	–	2/0	–	–	–	–	–	–	–	–	–	–
*C. minutissimus*(Zetterstedt, 1855)	(1)/0	–	–	–	–	–	–	–	–	–	–	(1)/0	–
*C. nubeculosus*(Meigen, 1930)	1/0	1/0	–	–	–	–	–	–	2(1)/0	3/0	4/0	–	4/0
Total number of females/males of other subgenera	69/0	1/0	2/0	0/0	0/0	0/0	0/0	0/0	11/0	3/0	4/0	1/0	4/0
*Dasyhelea* spec.	+	–	–	–	–	–	–	–	–	–	–	–	–
*Atrichopogon* spec.	–	–	–	–	–	–	+	–	–	–	–	–	–
*Forcipomyia* spec.	+	+	+	+	–	+	+	+	+	+	+	+	+

## Supplementary Materials

The following are available online at https://www.mdpi.com/1660-4601/17/14/5000/s1, Table S1: Detailed list of sites, locations, study regions and landscape/habitat types sampled for emerging adult biting midges.
