# Supplementary material for: Breeding Habitat Preferences of Major Culicoides Species (Diptera: Ceratopogonidae) in Germany
Source: Int J Environ Res Public Health. 2020 Jul 11;17(14):5000. doi: 10.3390/ijerph17145000 (PMC7400431; doi:10.3390/ijerph17145000)
Supplement: Supplementary file 1 [file ijerph-17-05000-s001.pdf]

International Journal of
Environmental Research and Public Health

MDPI

Table S1. Detailed list of sites, locations, study regions and landscape/habitat types sampled for emerging adult biting midges.

Trapping Site No.	Trapping Site Georeferences	Landscape/Habitat Type	Study Region in Figure 1	Location	Collection Year	Animal Husbandry	Number of Emergence Traps	Number of Collected Culicoids
1	52°45'48.13" N<br>14°18'30.49" E	Floodplain	A	River Oder<br>Güstebieser Loose	2008<br>2009<br>2010		10	82
2	52°23'39.28" N<br>13°53'54.04" E		C	River Spree<br>Freienbrink	2008		2	6
3	52°36'5.79" N<br>14°19'6.21" E		D	Backwater River Oder<br>Platkow	2008<br>2009<br>2010		2	13
4	52°46' .21" N<br>14°16'58.37" E	Meadows	A	River Oder<br>Güstebieser Loose	2007<br>2008<br>2009<br>2010		4	32
5	52°23'20.33" N<br>13°53'47.94" E		B	River Spree<br>Mönchwinkel	2008		2	4
6	52°59'24.92" N<br>12°54'50.79" E	Renaturation area/floodplain	E	Zippelsförde	2011		3	104
7	52°59'32.78" N<br>12°53'59.78" E			River Rhin<br>Zippelsförde	2005		1	21
8	50°3'20.35" N<br>8°2'16.06" E		F	Kisselmühle	2009		1	7
9	52°45'24.90" N<br>14°18'27.78" E	Mixed grassland/wetland without bushes and trees	A	River Oder<br>Güstebieser Loose	2008<br>2009<br>2010		6	59
10	52°22'49.29" N<br>13°47'30.61" E		C	River Spree<br>Freienbrink	2009		1	3
11	52°29'49.64" N<br>14°37'43.95" E		G	River Oder<br>Reitwein	2008<br>2009		4	19

12	52°45'24.48" N 14°18'24.94" E	Mixed grassland/wetland with bushes and trees	A	River Oder Güstebieser Loose	2008 2009 2010	2	57
13	52°22'27.26" N 13°47'46.03" E		C	River Spree Neu Zittau	2009	1	2
14	52°29'57.92" N 14°37'36.16" E		G	River Oder Reitwein	2008 2009	4	147
15	52°29'3.75" N 13°42'14.35" E		H	Schöneiche	2012	1	6
16	52°27'6.35" N 13°41'35.05" E	Swampy forest areas	H	Schöneiche	2008	1	11
17	52°33'1.48" N 14°4'25.15" E		I	Buckow		2	62
18	52°45'44.73" N 14°18'56.71" E	Bank of river	A	River Oder Güstebieser Loose	2007 2008 2009	2	75
19	52°32'30.40" N 14°4'40.18" E	Bank of lake	I	Däbersee Waldsieversdorf	2008 2009	4	4
20	52°32'19.05" N 14°5'0.02" E			Papillensee Waldsieversdorf		3	6
21	52°32'28.37" N 14°4'45.68" E	Reed belt	I	Däbersee Waldsieversdorf	2008 2009	2	71
22	52°32'13.52" N 14°4'53.25" E			Papillensee Waldsieversdorf		2	43
23	52°32'56.13" N 14°3'54.79" E	Bogland	I	Gartzsee	2009	2	115
24	52°37'44.26" N 14°18'21.03" E	Compost	D	Kiehnwerder	2007 2008 2009 2010	1	53
25	52°29'1.37" N 13°41'32.87" E		H	Schöneiche	2008 2010	1	11

26	52°32'32.15" N<br>14°4'37.18" E		I	Waldsieversdorf	1994<br>2008<br>2009		1	16
27	52°44'27.28" N<br>11°25'9.48" E		J	Jeetze	2010		1	3
28	52°44'52.94" N<br>14°20'23.92" E	Dung (animal holding)	A	River Oder<br>Güstebieser Loose	2008<br>2009	Cattle	4	134
29	52°45'17.27" N<br>14°19'58.86" E				2008<br>2009<br>2010	Cattle (cowpats)	10	95
30	52°23'15.53" N<br>13°52'56.37" E		B	Mönchwinkel	2008	Cattle<br>Sheep	2<br>2	28<br>15
31	52°23'31.83" N<br>13°53'42.30" E			River Spree<br>Mönchwinkel		Cattle/sheep	2	0
32	52°37'43.88" N<br>14°18'20.03" E		D	Kiehnwerder	2007<br>2008<br>2009<br>2010	Horse<br>Goat<br>Sheep<br>Horse/sheep<br>Horse/goat	2<br>1<br>1<br>1<br>1	15<br>0<br>17<br>121<br>176
33	52°36'48.25" N<br>14°18'4.01" E			Rosenthal		Cattle<br>Horse<br>Cattle/horse	2<br>2<br>2	7<br>28<br>92
34	52°35'42.64" N<br>14°18'37.58" E			Backwater River Oder<br>Platkow	2008<br>2009<br>2010	Duck<br>Pig<br>Sheep	1<br>1<br>2	21<br>5<br>0
35	52°59'33.72" N<br>12°54'30.29" E		E	Zippelsförde	2011	Sheep	1	30
36	50°3'15.91" N<br>8°2'18.48" E		F	Kisselmühle	2009	Llama/alpaca	1	174
37	52°29'3.03" N<br>13°42'12.98" E		H	Schöneiche	2012	Rabbit<br>Horse	1<br>1	31<br>20
38	52°29'4.40" N<br>13°42'20.26" E					Horse	1	13

39	52°29'2.50'' N<br>13°41'31.10'' E				2008<br>2010	Horse	4	22
40	52°44'26.95'' N<br>11°24'36'' E		J	Jeetze	2010	Duck<br>Chicken<br>Pig	1<br>1<br>1	1<br>13<br>1
41	52°32'30.91'' N<br>11°22'7.90'' E		K	Ackendorf	2012	Deer	1	21
42	52°36'49.24'' N<br>14°18'2.54'' E	Liquid manure (animal holding)	D	Rosenthal	2007<br>2008<br>2009<br>2010	Cattle	1	1
43	52°59'34.19'' N<br>12°54'28.99'' E		E	Zippelsförde	2011	Sheep	1	3
